# Influencing factors for the implementation of school-based interventions promoting obesity prevention behaviors in children with low socioeconomic status: a systematic review

**DOI:** 10.1186/s43058-024-00548-1

**Published:** 2024-02-12

**Authors:** Friederike Butscher, Jan Ellinger, Monika Singer, Christoph Mall

**Affiliations:** TUM School of Medicine and Health, Department Health and Sport Sciences, Technical University of Munich, Georg-Brauchle-Ring 60/62, 80992 Munich, Germany

**Keywords:** Health equity, Implementation, Obesity, Systematic review, CFIR, Qualitative review, School-based interventions, Children, Facilitators

## Abstract

**Background:**

Health inequity (HI) remains a major challenge in public health. Improving the health of children with low socioeconomic status (SES) can help to reduce overall HI in children. Childhood obesity is a global problem, entailing several adverse health effects. It is crucial to assess the influencing factors for adoption, implementation, and sustainment of interventions. This review aims to identify articles reporting about influencing factors for the implementation of school-based interventions promoting obesity prevention behaviors in children with low SES. It aims to critically appraise the articles’ quality, assess influencing factors, categorize and evaluate them, and to discuss possible implications.

**Methods:**

A systematic search was conducted in 7 databases with the following main inclusion criteria: (1) school-based interventions and (2) target group aged 5–14 years. The Consolidated Framework for Implementation Research, its five domains (intervention characteristics, inner setting, outer setting, characteristics of individuals, process) along with 39 categories within these domains were used as deductive category system for data analysis. We grouped the articles with regard to the characteristics of the interventions in *simple* and *complex* interventions. For each domain, and for the groups of *simple* and *complex* interventions, the most commonly reported influencing factors are identified.

**Results:**

In total, 8111 articles were screened, and 17 met all eligibility criteria. Included articles applied mixed methods (*n*=11), qualitative (*n*=5), and quantitative design (*n*=1). Of these, six were considered to report *simple* interventions and eleven were considered to report *complex* interventions. In total, 301 influencing factors were assessed. Aspects of the *inner setting* were reported in every study, aspects of the *outer setting* were the least reported domain. In the *inner setting*, most reported influencing factors were *time* (*n*=8), *scheduling* (*n*=6), and *communication* (*n*=6).

**Conclusion:**

This review found a wide range of influencing factors for implementation and contributes to existing literature regarding health equity as well as implementation science. Including all stakeholders involved in the implementation process and assessing the most important influencing factors in the specific setting, could enhance implementation and intervention effectiveness. More empirical research and practical guidance are needed to promote obesity prevention behaviors among children with low SES.

**Registration:**

CRD42021281209 (PROSPERO)

**Supplementary Information:**

The online version contains supplementary material available at 10.1186/s43058-024-00548-1.

Contributions to the literature • The in-depth application of the Consolidated Framework for Implementation Research (CFIR) in this review facilitates comparability and transferability between findings of this review and other research findings.

• This review places a focus on the implementation of obesity prevention interventions for children with low socioeconomic status, thus expanding the literature related to health equity.

• The synthesis of the included papers in this review provides guidance that specifically addresses intervention developers, school staff, and researchers, respectively, and can therefore help to inform the selection of implementation strategies and planning.

## Background

“Implementation Science could, quite literally, put health equity back on the fast track.” Beryne Odeny [1]

Health inequalities are the difference between the health statuses of groups of people, they exist within and between populations [2]. One example is the difference in life expectancy within a population (e.g., between men and women) as well as between populations (e.g., between women in one population and another) [2]. Determinants of health include for example, fixed determinants like genes and age, and modifiable determinants like the individual lifestyle, social networks and broader aspects like the cultural, social, and physical environment [3]. Furthermore, interactions between determinants can occur, as for example, the wider sociocultural environment is linked to social norms, and social norms impact in turn individual lifestyles [3]. The social determinants of health, all (theoretically) modifiable determinants, are a powerful driver for health (in)equalities [4–6]. Under certain conditions, we no longer speak of health inequalities but of health inequity (HI) [7, 8]. If health inequalities are avoidable and unfair [9], then we speak of health inequity. HI arises due to differences in opportunity, more specifically the unequal distribution of the social determinants of health, such as income, wealth, and access to health care [6, 10]. Therefore, HI is social injustice in health [8] or vice versa: “Equity in health means that people’s needs guide the distribution of opportunities for well-being” [10].

HI follows a social gradient, as groups with a low socioeconomic status (SES) have poorer health (e.g., higher mortality and morbidity) than groups with high SES [6, 7, 11]. SES is a commonly used proxy for social determinants of health [12, 13], as SES is a multidimensional concept and incorporates several socioeconomic factors. It can be described by past or current income, family wealth, educational level, occupation, and social standing within the community [14].

It is especially important to protect children’s health, as they have less control over their health and the circumstances influencing it than adults [15], as adults form the environment children live in (e.g., at home or school). Negative health influences in childhood can lead to health consequences throughout life [16, 17]. Being overweight in childhood, for example, is associated with also being overweight as an adult [18], and diverse adverse health effects, such as cardiovascular diseases or mental disorders, can result from overweight and obesity in childhood [19, 20]. The prevalence of obesity in children is increasing globally [21, 22], and therefore, it is important to develop and implement interventions addressing childhood obesity.

In industrialized countries, childhood obesity exhibits HI: This is reflected, for example, in the fact that a low SES is associated with higher rates of obesity among children [23].

Furthermore, due to societal processes, low SES and poor health implicate and maintain each other [3, 8, 24, 25]. In their model of child health inequalities, Pearce et al. [15] described those societal processes and showed that low SES and low child health status are in a mutually reinforcing cycle, conditioning and maintaining each other.

How child health status and SES condition and maintain each other, and therefore how HI is maintained, is described with five mechanisms. (I) *Social stratification* refers to all social structures that influence the SES of children (e.g., growing up in a low-income household compared to a high-income household). (II) *Differential exposure* describes how children living under different SES are exposed to different levels of health risks (e.g., living in a noisy/polluted area, because rents are lower in such areas). (III) *Differential vulnerability* means that exposure to a greater number of health risks and their interaction may increase vulnerability to adverse health outcomes (e.g., job loss of parents causes more mental health burden in a low-income household compared to a high-income household). The (III) *differential vulnerability* caused by the greater number of health risks influences the rest of the life, and thus also the future SES. This influence of (III) *differential vulnerability* on SES is referred to as (IV) *differential consequences* (e.g., child in a noisy household with mentally stressed parents could lead to a challenging learning atmosphere and could result in bad grades in school). Through these described four mechanisms, (V) *further social stratification* emerges (e.g., bad grades in school lead to a lower-payed job). From the mechanism of (V) *further social stratification*, it becomes clear that the SES not only has an impact on health, but that low SES and low child health status are in a mutually reinforcing cycle, conditioning and maintaining each other [15].

One suitable entry point to address (II) *differential exposure* and (III) *differential vulnerability* are health-promoting interventions. Health-promoting interventions can mitigate (IV) *differential consequences* and therefore mitigate (V) *further social stratification.* Health-promoting interventions that improve the health status of children can therefore help to reduce HI in children [15].

Many health-promoting interventions take place in schools, as in the school setting almost all children in society can be reached [26]. This also applies to obesity prevention interventions [27], for now moderate evidence has been found for school-based combined diet and physical activity (PA) interventions [28–30]. Furthermore, it is important to implement those interventions in real-world settings, as the implementation of an intervention influences its effectiveness [31, 32]. Improving the reach and the adoption, delivery, and sustainment of effective interventions is the aim of implementation science [33]. Because several factors influence the speed and extent of the adoption, uptake, and use of an intervention (e.g., characteristics of the intervention like complexity or contextual factors like built environment) [34], a suggested first step in the implementation process is the identification of those influencing factors in order to address them [35].

The influencing factors for the implementation of interventions have been assessed in the school setting, both for PA-promoting interventions [32] and for interventions to promote PA and reducing sedentary behavior [36]. Barriers and facilitators were assessed for the sustainment of health behavior interventions in schools and childcare settings [37], for PA during school lessons [38], and for the provision of fruit and vegetable in kindergartens and schools [39].

Those reviews [32, 36–39] present important results, but none of those reviews distinguished between different SES, although this factor is an important differentiator every study should take into account to approach health equity [40]. Furthermore, none of the existing reviews assessed the implementation of interventions addressing the combination of the two leading domains of behaviors in obesity development, namely, PA and nutrition [41], in the school setting. From these considerations, it seems essential that factors influencing the implementation of school-based interventions be systematically assessed to promote obesity prevention behaviors for children with low SES. These findings can help improving the understanding of specific needs, to guide practice, to improve implementation, and therefore, to enhance the sustainment of effective interventions. Effective interventions can contribute to prevent obesity, increase the health of children (with low SES) and reduce *further social stratification*. This could contribute to reducing HI in children. Therefore, this review aimed to identify articles reporting about influencing factors for the implementation of school-based interventions promoting obesity prevention behaviors for children with low SES, to assess the methodological quality of the identified articles, to categorize and evaluate reported influencing factors, to analyze differences of reported influencing factors regarding *simple* and *complex* interventions, and to discuss possible implications.

## Methods

We identified, critically appraised, and summarized the published evidence on influencing factors for the implementation of school-based interventions promoting obesity prevention behaviors for children with low SES by means of a systematic review in accordance to PRISMA guidelines [42], the PRISMA Checklist can be found in the Additional file 1. This review was previously registered at PROSPERO (ID: CRD42021281209).

### Information sources and searches

The databases Scopus, PubMed, ERIC, SportDiscus, PsychArticles, Education Source, and SocINDEX were searched for relevant articles. The terms shown in Table 1 were used to construct the search term, following database specifications (see Additional file 2). There were no limitations with respect to the publication date of the articles, as no systematic review with the same aim had previously been conducted. The database search was completed on July 2, 2021. To ensure actuality, we conducted an update search on March 29, 2023.

**Table 1:**
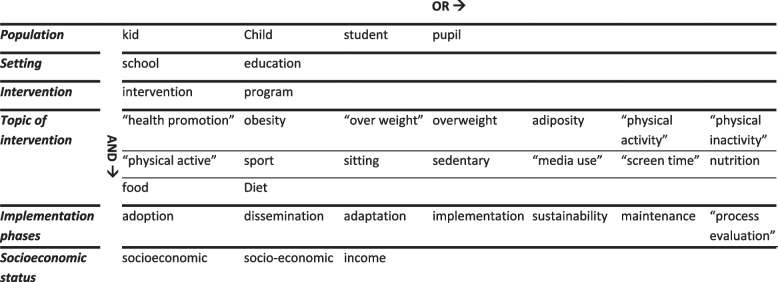
Search term

### Eligibility criteria

We adopted the following eligibility criteria:

- Regarding the design, the article had to be an implementation evaluation or process evaluation study, or a hybrid process-effectiveness study as described by Curran et al. [43].
- Qualitative, quantitative and mixed methods studies were eligible for inclusion.
- The article had to investigate an intervention promoting obesity prevention behaviors (e.g., promotion of PA, promotion of health nutrition).
- The intervention reported had to address children aged 5–14 years exclusively. The youngest age for beginning primary school is 5 years [44] and 14 years is the last year of childhood, before entering the youth category [45].
- The intervention reported had to be conducted in a school setting.
- The intervention reported had to be conducted in an area with population of low SES or address children with low SES in particular.
- The article had to report influencing factors for the implementation of the intervention in their results section regarding children with low SES.

### Operationalization of SES

Measuring the SES of children is challenging as they do not have their “own” SES. Parental income, parental education, and parental occupation are often used to measure children’s SES [13, 46]. More broadly, children’s SES can be for example measured by the socioeconomic characteristics of the neighborhood [47]. Consequently, the parameters described previously that apply to the parents and their SES would also be applied to the child in question. These measures are correlated but not interchangeable [46]. Aggregated measures are also used to establish SES for school population or for regions or districts. Those aggregated measures would be drawn from administrative data and therefore depend on the institutional understanding of SES [46] and the availability of data. For this review, study authors reporting that low SES children had been focused upon in their research was considered sufficient, and a range of criteria and measures used to assess SES were accepted. The information on the criteria used to define low SES was extracted from the articles and is shown in the “Results” section.

### Screening process

After deduplication, two reviewers (FB and JE) independently screened the articles on title and abstract level and in a second step on full text level using the software Rayyan to determine inclusion [48]. Conflicts were discussed and resolved between the reviewers. Additionally, all articles included in the review by Cassar et al. [36] were screened at full text level, as that review had a very similar aim, with the exception of the focus on SES.

### Data extraction and synthesis

Article title, year of publication, country, aim of the evaluation, outcome variables assessed, means of data collection, criteria for low SES, and description of the intervention were extracted into one file (see Additional file 3) by MS and FB from each article. After the screening process, two reviewers (FB and JE) extracted barriers and facilitators for implementation from the results section of the articles into an Excel file. FB and JE extracted data from two articles independently and then matched their results through discussion. The remaining articles were split between FB and JE for data extraction. When any uncertainties arose about which details to extract from an article, a second reviewer extracted data from the same article and then any discrepancies were resolved through discussion.

The Excel file with extracted data was loaded into MAXQDA [49] for qualitative content analysis. The analysis was guided by the *Consolidated Framework of Implementation Research* (CFIR) [50]. This comprises the five domains (all expanded in detail below), along with 39 categories within these domains [50]. *Intervention characteristics* focus on the features of the intervention itself, for example, the source of the intervention or the design and packaging. *Inner setting* includes aspects of the setting, in which the intervention is being implemented, for example, the extent to which the intervention is prioritized compared to other activities within the setting. *Outer setting* is the setting, in which the *inner setting* exists in terms of structural, political, and cultural contexts, for example, policies, that must be adhered to. *Characteristics of individuals* include characteristics of people involved with the intervention, for example, their motivation or knowledge about the intervention. *Process* include all strategies and processes of implementing the intervention, for example, feedback processes, for example reflecting and evaluating on the quality during the implementation [50].

The 39 categories within these five domains from CFIR were used to deductively develop a category system for qualitative content analysis of the data. Each sense unit was coded into only one category. If reasonable due to different aspects within one category, subcategories were developed inductively. FB and JE coded 25% of the data independently, then the codes were reconciled, and the rest of the data was coded by FB. In the next step, all coded segments for each category were reviewed by FB. For a clear differentiation between certain categories for this review, additional specifications were developed (see Additional file 4) and, if necessary, the coding of the segments was adjusted according to the differentiations made between the categories. To test the final and refined category system, two reviewers (JE and a student assistant) coded 50% in total of the data again. The double-coded data were compared, and differences were discussed and resolved. All categories that caused more than one disagreement on the segment level were reviewed again for all data by FB. In the last step, all categories were reviewed. Content-related subcategories were developed inductively and coded again by a student assistant, and any disagreements were discussed and resolved.

For each of the five CFIR domains, the average number of articles per (sub)category (level above the barrier/facilitator) was calculated, by taking the total number of articles reporting on the categories within the domain and dividing it by the number of categories within the domain. This means, one articles could have been counted twice (or more), when it reported on two (or more) categories within the domain. For example, the domain of Intervention Characteristics includes 10 categories, and summed up, 33 articles reported factors related to this domain (see additional file 8). Dividing the total of 33 articles by the 10 categories results in an average article rate of 3.3 per category for this domain. In the “Results” section and in Figs. 2, 3, 4, 5, 6, 7, and 8 only (sub)categories are presented, which were reported above-average frequency for each domain respectively.

We grouped and then compared the articles with regard to the characteristics of the interventions, following the definition for complex interventions by Craig et al. [51]. If the intervention met two out of the three following aspects, it was considered to be *complex*, and otherwise it was considered to be *simple*: the intervention [1] addressed more than one obesity prevention behavior, [2] consisted of more than one component (e.g., classroom activities and teacher training), and [3] included parental involvement. We analyzed the most frequently reported influencing factors within those groups of *simple* and *complex* interventions.

### Methodological quality assessment

We assessed the methodological quality of the articles using the Mixed Methods Appraisal Tool (MMAT) Version 2018 [52, 53]. The MMAT allows the rating of quantitative and qualitative articles in the two separate corresponding categories, and mixed methods articles are rated in both, as well as an additional third mixed methods category.

Two reviewers (FB and JE) individually assessed the methodological quality of three articles, and the results were discussed with a third reviewer (CM). All of the remaining articles were split between two reviewers (FB and JE), and the methodological quality was individually assessed. If any uncertainties arose regarding the methodological quality of any particular article, it was assessed and evaluated by the reviewers individually, results of the individual assessments were discussed and the uncertainties resolved. No overall score was calculated, as recommended by the authors of MMAT [52].

## Results

### Study selection

**Fig. 1:**
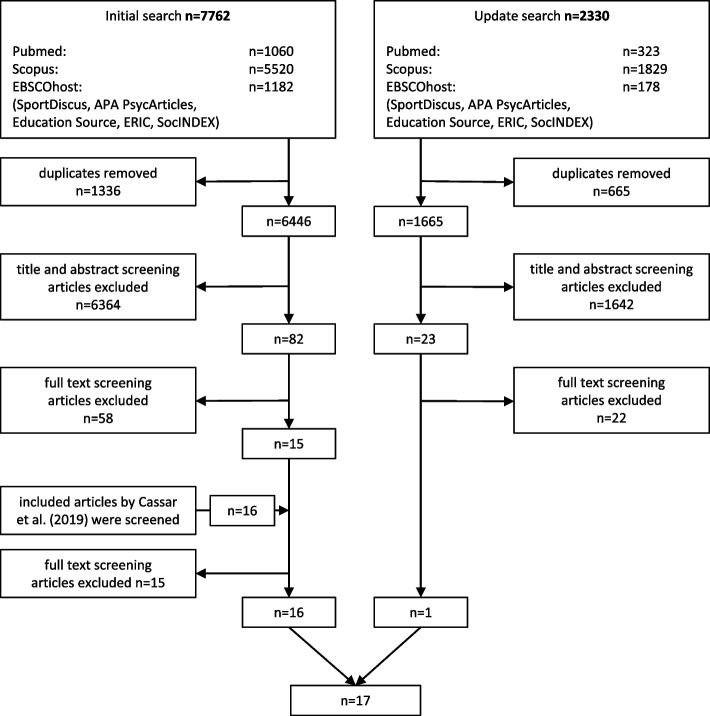
Flow chart of screening

In total, 8111 articles were screened, 15 articles were identified as meeting all eligibility criteria from the initial search and screening of 6446 articles. Screening the articles included by Cassar et al. [36], one additional article met all eligibility criteria. The update search and screening of 1665 articles resulted in one additional article eligible for inclusion. In total, 17 articles were included in this systematic review [54–70] (see Fig. 1).

### Study characteristics

Additional file 3 presents detailed information on the included articles, such as their aim and information on the intervention reported. The aims of eleven articles was to assess the implementation of the intervention and additional influencing factors for implementation ([54, 56, 57, 63–65, 68, 70, 59–61]). Five articles only assessed influencing factors for implementation [55, 62, 66, 67, 69], and one assessed the influencing factors for implementation, as well as the effectiveness of the intervention [58]. The articles applied mixed methods (*n*=11), qualitative (*n*=5), and quantitative design (*n*=1). Data collection methods included interviews [54, 56, 57, 60, 64, 65, 68, 69], questionnaires [54, 56–60, 64, 65, 68, 70], focus groups [55, 61–63, 66, 67], observations [54, 60, 64, 69], document analyses [57, 68, 69], app usage [61], run tests [63], and accelerometers [70]. Data was collected from teachers or other school staff (head teachers, physical education teachers, program leaders) [54–60, 63–67, 69, 70], students [54, 57, 58, 60–64, 69, 70], parents [64, 66, 67], and externals like local sports coordinators [57, 69].

The interventions reported by the articles promoted PA [57–59, 63, 68, 70], healthy nutrition [60, 64, 65], PA and healthy nutrition [54, 55, 62, 66, 67, 69], and PA, healthy nutrition and reducing screen time [61] and PA, healthy nutrition, healthy sleep, and reduce screen time [56]. In total, 15 independent interventions were reported, and three articles reported on the same intervention [62, 66, 67]. Five interventions were conducted in the USA [55, 56, 60, 68, 70], four in the Netherlands [54, 57, 64, 69], two in Canada [58, 59], one in Australia [61], one in Germany [65], one in Sweden [62, 66, 67], and one in the UK [63]. Six articles were considered reporting on *simple* interventions [58, 59, 63, 65, 68, 70], and eleven articles were considered reporting on *complex* interventions [54, 55, 57, 60–62, 64, 66, 67, 69] (see Additional file 3). Most frequent intervention components included classroom activities [55–57, 60, 62, 66, 67, 69], physical activity lessons [54, 58, 63, 69, 70], financial support or materials provided to schools [54, 56, 59, 61, 64, 65, 68], and parental information [57, 58, 60, 62, 64, 66–68].

### Quality assessment results

Additional file 5 presents the ratings of the methodological quality assessment. Four [55, 62, 66, 67] of the five qualitative articles received a “yes” for all criteria, and one article received a “no” for the criterion “Is the interpretation of results sufficiently substantiated by data?” [69]. The only quantitative article received a “can’t tell” for the criterion “Is the sampling strategy relevant to address the research question?” [65]. Of the eleven mixed methods articles, five [54, 58, 61, 63, 68] received a “yes” for all qualitative criteria, whereas only one of them received all quantitative criteria rated with “yes” [68]. Six [54, 56, 57, 59–61] of the eleven mixed methods articles received a rating of “yes” for all mixed methods criteria, one article [63] received a “no” for the criterion “Is there an adequate rationale for using a mixed methods design to address the research question?”. None of the mixed methods articles received only “yes” ratings for all criteria. In the mixed methods articles, the qualitative items rated lower than the qualitative articles.

### Influencing factors for implementation

In the following, selected results are presented to answer the research question what the influencing factors for the implementation of school-based interventions promoting obesity prevention behaviors for children with low SES are.

Table 2 presents all included articles and their reporting of influencing factors in the five domains of CFIR, as well as the assignment to the groups of *simple* or *complex* interventions. The *inner setting* was reported in all articles (*n*=17), and the least reported domain was the *outer setting* (*n*=8). The *outer setting* was only reported in the group of *complex* interventions. Every article reported influencing factors in at least three different domains. In the 17 articles, 301 influencing factors were found across 89 (sub)categories, consisting of 35 categories (coming from the deductively developed categories) from CFIR and 55 categories (coming from the inductively developed categories). Of the 39 original CFIR categories, four categories were not reported at all. Additional file 6 presents all identified influencing barrier and facilitators for all (sub)categories for each domain. Additional file 7 presents the most commonly reported (sub)categories in the group of *simple* and *complex* interventions for each domain.

**Table 2:** Reported CFIR domains in included articles

	Article																	
CFIR domain (number of (sub-)categories in domain)	Bartelink [54]*	Bauer [55]*	Blaine [56]*	de Meij [57]*	Gadais [58]°	Gosslin [59]°	Lepe [60]*	Lubans [61]*	Malek [62]*	Marchant [63]°	Martens [64]*	Muckelbauer [65]°	Norman (a) [66]*	Norman (b) [67]*	Steward [68]°	Verjans-Janssen [69]*	Wright [70]°	
Intervention characteristics (10)	x		x	x	x		x	x	x	x	x	x	x	x	x	x	x	15
Inner setting (26)	x	x	x	x	x	x	x	x	x	x	x	x	x	x	x	x	x	17
Outer setting (8)	x	x	x	x		x							x	x		x		8
Characteristics of the individual (14)		x	x	x	x	x	x	x	x	x	x	x	x	x	x	x		15
Process (16)	x	x	x	x	x	x	x	x	x	x	x		x	x	x	x	x	16
	4	4	5	5	4	4	4	4	4	4	4	3	5	5	4	5	3	

*Group of *complex* interventions

°Group of *simple* interventions

**Fig. 2:**
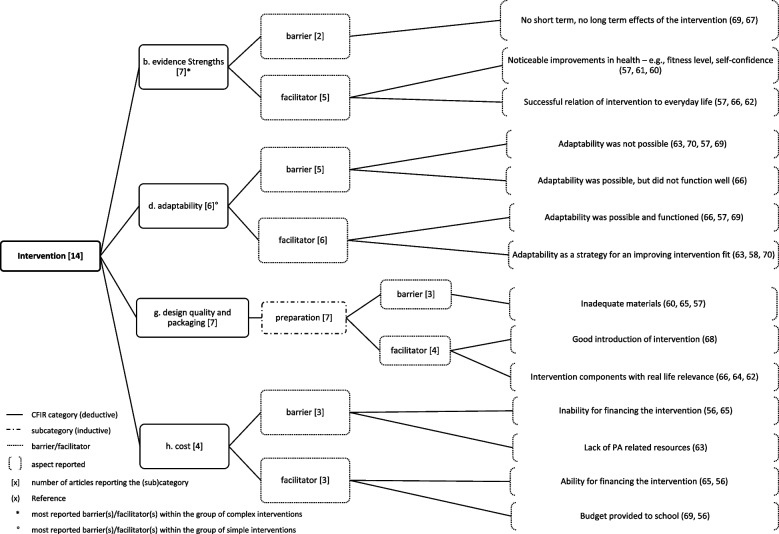
Intervention characteristics and most reported (sub)categories

**Fig. 3:**
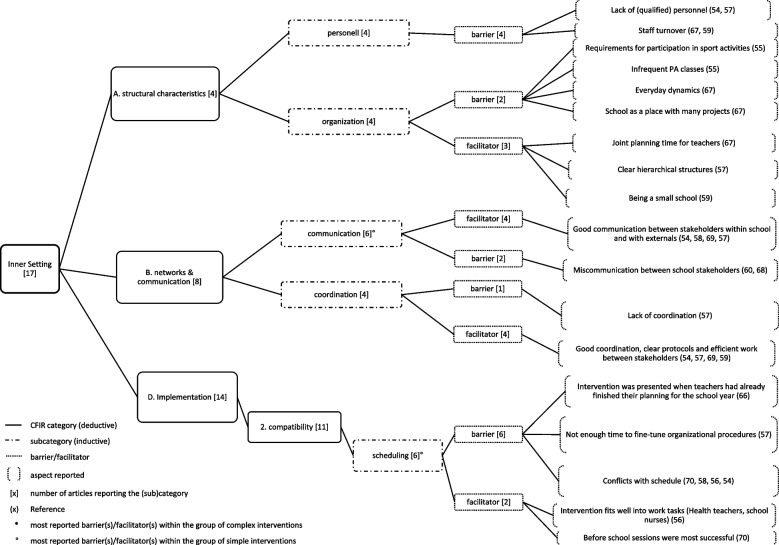
Inner setting (1) and most reported (sub)categories

**Fig. 4:**
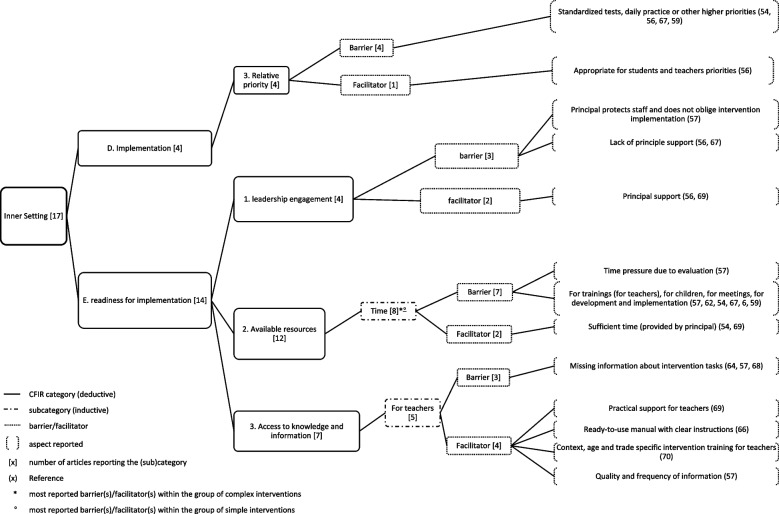
Inner setting (2) and most reported (sub)categories

**Fig. 5:**
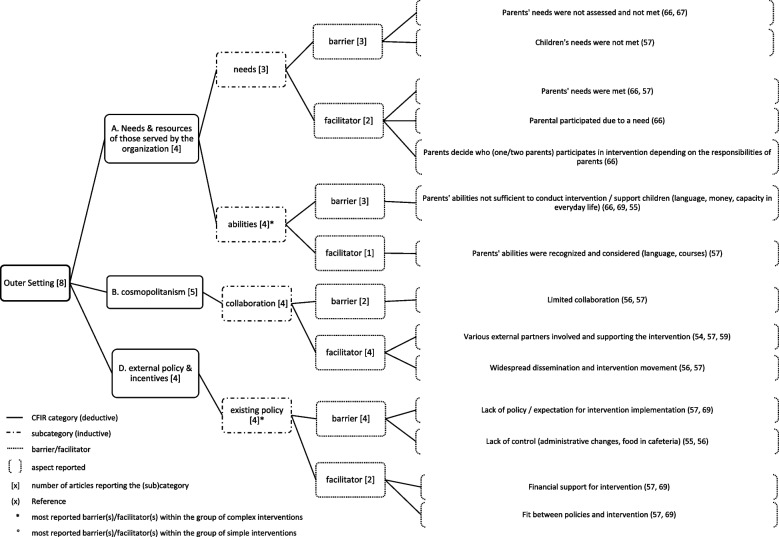
Outer setting and most reported (sub)categories

**Fig. 6:**
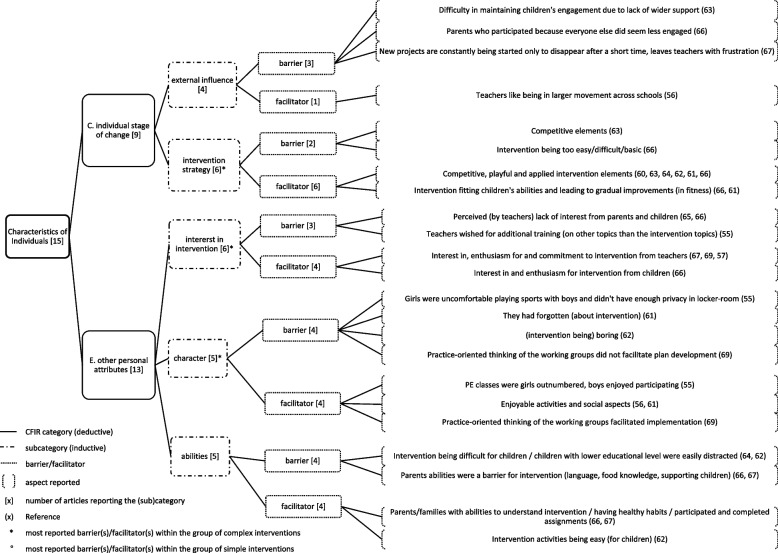
Characteristics of Individuals and most reported (sub)categories

**Fig. 7:**
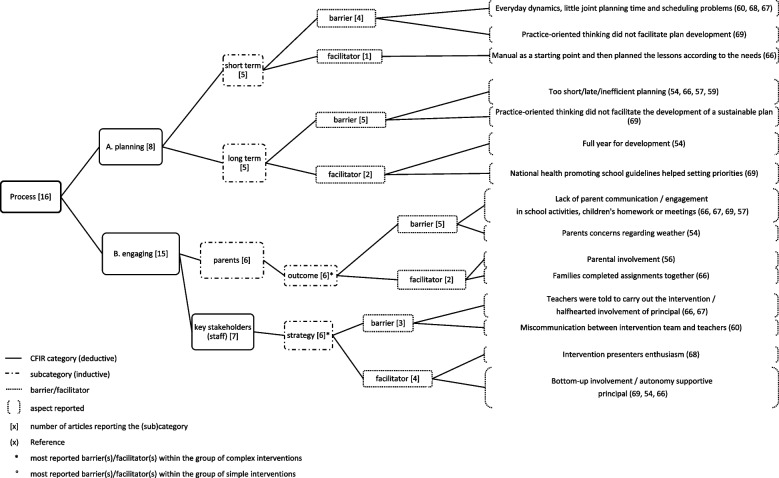
Process (1) and most reported (sub)categories

**Fig. 8:**
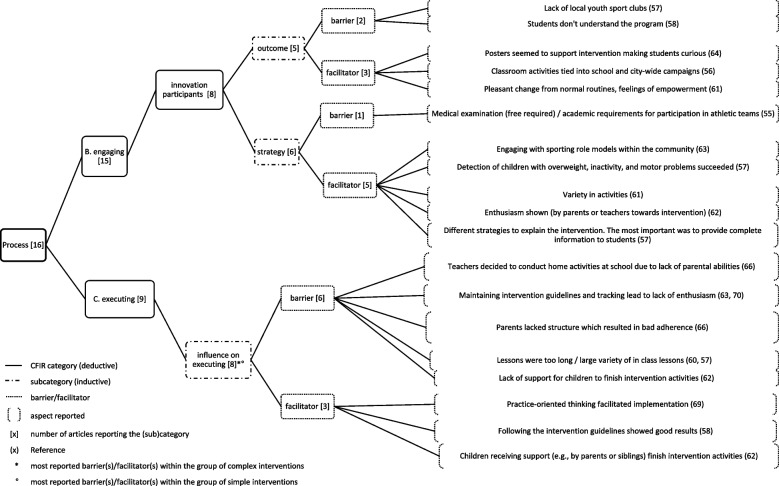
Process (2) most reported (sub)categories

The results for each domain are presented below, with the number of articles reporting on the domain respectively, with the most reported influencing factors within each domain, and within the group of *simple* and *complex* interventions. Figs. 2, 3, 4, 5, 6, 7, and 8 show (sub)categories with above-average frequency for each domain, as well as the reported barriers and facilitators in those (sub)categories. Furthermore, the most reported barrier(s) or facilitator(s) for each group of interventions is marked. Additional file 8 presents all (sub)categories for each domain, and all the number of articles reporting the relevant barriers and facilitators.

### Intervention characteristics

*Intervention characteristics* were reported in 14 of the included articles. The average number of articles reporting on the 10 (sub)categories was 3.3 per category. In total, four (sub)categories were reported with an above-average frequency and are displayed in Fig. 2. The most reported influencing factors are described here.

*Evidence strengths (perception of the quality and validity of evidence supporting the belief that the intervention will have desired outcomes)* (*n*=7) was reported as a barrier, because no short- or long-term effects of the intervention (*n*=2) were seen. As facilitating for the implementation of the interventions, noticeable improvements in health (e.g., in the fitness level or self-confidence of children) (*n*=3) and successful linking of intervention topics with the children’s everyday life (*n*=3) were reported.

The *preparation (perception of presentation and quality of intervention materials)* (*n*=7) of the intervention was reported as facilitator as good introduction to the intervention (*n*=1) and intervention components with real-life relevance (*n*=4) (e.g., hands-on sessions, real-life relevance of intervention components). In *n*=3 articles, barriers as inadequate intervention materials (e.g., wordiness of lessons) were reported.

Within the group of *simple* interventions, *adaptability (the degree to which an intervention can be adapted, tailored, refined, or reinvented to meet local needs)* (*n*=3) was the most reported influencing factor as adapting the interventions as strategy for an improved fit (e.g., by trying different times during school day for extra PA lessons) (*n*=3) facilitated implementation. Within the group of *complex* interventions, *evidence strengths* (*n*=7) was the most reported influencing factor. Only studies reporting on complex interventions reported on *evidence strengths.*

### Inner setting

*Inner setting* was reported in all 17 included articles. The average number of articles reporting on the 26 (sub)categories was 3.1 per category. In total, nine (sub)categories were reported with an above-average frequency and are displayed in Figs. 3 and 4. The most reported influencing factors are described here.

The most reported influencing factor was *time* (*available time for implementation activities*) (*n*=8), with sufficient time (*n*=2) facilitating and insufficient time hindering (*n*=6) (e.g., for meetings, for training, for the children, for implementation or insufficient time due to the evaluation timeline) was also reported as hindering implementation.

*Scheduling (perceived compatibility of intervention activities with workflows)* (*n*=6) was reported as a barrier (*n*=6), due to conflicts with scheduling (*n*=4), insufficient fine-tuned organizational procedures (*n*=1), and the school year was already planned, when the intervention was introduced (*n*=1). As a facilitator, *scheduling* was reported (*n*=2) in terms of a good fit of intervention in the work tasks (*n*=1) and scheduling the intervention activity before school was successful (*n*=1).

In the subcategory of *communication (nature and quality of formal and informal communication with externals and within organization)* (*n*=6), good communication between stakeholders within school and with externals was reported as a facilitator (*n*=5) and miscommunication between school stakeholders (*n*=2) was considered a barrier for implementation.

Within the group of *simple* interventions, those *time*, *scheduling*, and *communication* (*n*=2) were the most reported influencing factors, and within the group of *complex* interventions, *time* (*n*=6) was the most commonly reported influencing factor.

### Outer setting

*Outer setting* was reported in eight of the included articles. The average number of articles reporting on the 8 (sub)categories was 2.8 per category. In total, four (sub)categories were reported with an above-average frequency and are displayed in Fig. 5. The most reported influencing factors are described here.

*Abilities (abilities of parents)* (*n*=4) was reported as a barrier, because of lack of sufficient abilities among parents to conduct intervention / support children (*n*=3), whereas recognized and considered parents’ abilities (*n*=1) facilitated implementation.

*Collaboration (status-quo of collaborations)* (*n*=4) was reported as barrier due to limited collaboration (*n*=2) and as facilitator, because of various external partners supporting the intervention (*n*=3) and widespread dissemination and intervention movement (*n*=2).

*Existing policy (a broad category including policy and regulations, external mandates, recommendations)* (*n*=4) were reported as barriers due to lack of policy/expectation from external for intervention implementation (*n*=2) and lack of control (e.g., over administrative changes, food in cafeteria) (*n*=2). Financial support for the intervention (*n*=2) and fit between policies and intervention topics (*n*=2) were reported as facilitators for implementation.

One of the articles within the group of *simple* interventions reported one influencing factor in the *outer setting*, therefore the subcategory *collaboration* (*n*=1) is the only and the most reported influencing factor in the groups of *simple* interventions as presence of partnerships facilitating the implementation (*n*=1). Within the group of *complex* interventions, *abilities* and *existing policies* (*n*=4) were the most reported influencing factors (see paragraphs above).

### Characteristics of individuals

*Characteristics of individuals* were reported in 14 of the included articles. The average number of articles reporting on the 14 (sub)categories was 3.4 per category. In total, five (sub)categories were reported with an above-average frequency and are displayed in Fig. 6. The most reported influencing factors are described here.

*Intervention strategy (strategies aiming to engage and motivate participants)* (*n*=6) was reported as a barrier due to competitive elements of the intervention for children activities (*n*=1) and difficulty or ease of the intervention tasks (*n*=1). As competitive, playful and applied intervention components (*n*=5), and fitting the intervention to children’s abilities and leading to gradual improvement in fitness (*n*=2) were reported as facilitators.

*Interest in intervention (interest of stakeholders in the intervention components/topics)* (*n*=6) was reported as a barrier, due to a lack of interest by parents and children in intervention (*n*=2) and teachers’ wish for additional training on topics other than the intervention topics (*n*=1). Interest in, enthusiasm for, and commitment to the intervention from children (*n*=1) and from teachers (*n*=3) facilitated implementation.

Within the group of *simple* interventions, *effect of stage (the effect of the individual stage of attitude towards an enthusiastic and sustainable usage of the intervention)* (*n*=2) as disengaged teachers resulted in disengaged students and vice versa (*n*=2) was the most reported influencing factor hindering implementation. Within the group of *complex* interventions, the most reported influencing factor was *character (other character traits influencing implementation)* (*n*=5) as, for example, forgetting about intervention (*n*=1) as barrier, or girls feeling more comfortable in activities where they outnumbered boys (*n*=1) as facilitator for implementation.

### Process

The *process* domain was reported in 15 of the included articles. The average number of articles reporting on the 16 (sub)categories was 3.8 per category. In total, seven (sub)categories were reported with an above-average frequency and are displayed in Figs. 7 and 8. The most reported influencing factors are described here.*Influence on executing (aspects affecting the execution of the intervention)* (*n*=8) was reported as lack of support for children to finish intervention activities (*n*=1), in-class lessons that were too long or too diverse (*n*=2), parents lacking structure regarding the intervention (*n*=1), teachers conducting home activities in school due to lack of parental ability (*n*=1) and sticking to intervention guidelines leading to lack of enthusiasm (*n*=1). However, practice-oriented thinking of stakeholders (*n*=1), children receiving support to finish intervention activities (*n*=1), and following the intervention guidelines (*n*=1) were reported as facilitators.

Within the group of *simple* interventions, *influence on executing* (*n*=3) was the most reported influencing factor as sticking to intervention guidelines and tracking lead to reduced enthusiasm (*n*=2) and hindered implementation. Within the group of *complex* interventions, the *outcome of parental engagement* (*n*=6) as for example, lack of parental communication and engagement (*n*=4) hindered implementation, and *influence on executing* (*n*=5) were the most reported influencing factor.

## Discussion

This review identified, categorized, and evaluated influencing factors for the implementation of school-based interventions promoting obesity prevention behaviors in children with low SES. We identified 301 influencing factors reported in 17 articles across 89 (sub)categories in the five domains of CFIR. The articles examined were grouped in a set of six *simple* and eleven *complex* interventions.

Aspects of the *inner setting* (also referred to as *organizational*) were reported in every article, and aspects of the *outer setting* (also referred to as *context*) constituted the least reported domain. These findings are consistent with the results of comparable reviews that assessed influencing factors on interventions promoting PA [32, 38] and reducing sedentary behavior in school settings [36], as well as the sustainment of health behavior interventions in school settings and childcare services [37]. Comparable reviews [32, 36–38] did not specifically address children with low SES. Although the present review and comparison reviews analyzed different target groups, the results are still comparable in the domain level. Therefore, one can consider that the presented results on this higher level are independent of the SES of children.

The *inner setting* is the most comprehensive domain of the CFIR, which likely have led to the accumulation of identified influencing factors. Although the *outer setting* is of great importance for implementation [31, 71–73], it is the least reported domain.

Due to the huge variety of identified influencing factors, in the following sections, selected aspect, relevant for intervention developers, school staff, and researchers, will be discussed. All findings can be found in the supplementary files and we are happy to provide additional information upon request.

### Influencing factors—for intervention developers

We grouped the articles in a set of *simple* and *complex* interventions, because *complex* interventions might entail a wider range of influencing factors than *simple* interventions (e.g., implementing an additional sport lessons, as *simple* intervention, is influenced by less factors than implementing additional sport lessons and healthy lunchboxes, as *complex* intervention). Furthermore, there is moderate evidence that *complex* interventions are more effective than *simple* interventions [28–30]. Comparing the groups of *simple* and *complex* interventions, one of the most reported influencing factors was *executing* in both groups.

*Executing* (also referred to as *fidelity* [74]) defined as *carrying out or accomplishing the implementation according to plan* [50] and *adaptability* (*the degree to which an intervention can be adapted, tailored, refined, or reinvented to meet local needs*) are highly connected. *Adaptability* was indeed the most reported influencing factor within the group of *simple* interventions.

There are examples in both groups of interventions for *adaptability* of the interventions, adaptations made, and their influence on *executing*. For example, in the group of *simple* intervention for example, having running routes inside, instead of outside, caused challenges [63]. Furthermore, sticking to the original principles and monitoring the intervention can lead to lack of enthusiasm [59, 68]. In the group of *complex* interventions, for example, lack of support for children to complete intervention activities [62, 66] or large variation of the time spent on activities [60] were reported as barriers for implementation. Children receiving support [69] and practice-oriented thinking by the executers [69] facilitated implementation. The examples mentioned in the groups of *simple* and *complex* intervention are different, though we cannot evaluate what reason for this is.

*Adaptability* is important to meet local needs, but adaptations mostly decrease the *executing* of an intervention. Adaptations are quite relevant for implementation [75] and *executing* is often used as outcome for measuring the degree of implementation [74]. To analyze the influence of *adaptability*, adaptations made, and *executing* on health outcome, it is important to document and consider both [75].

We grouped the articles according to the intervention characteristics, following the criteria for *complex* interventions by Craig [51]. This is one option for grouping the interventions, it could be argued that every intervention itself can be considered a complex intervention, following different criteria (e.g., synergies between intervention components, degree of flexibility, and multiplicity of mediators or moderators) [76]. Furthermore, if the intervention itself is not complex, one could argue that the school setting, with its context and stakeholders, and the interactions between them certainly can be considered complex (regardless of how simple or complicated the intervention is) [77, 78]. Those different options for grouping interventions and furthermore different perspectives regarding school as a setting reflect on the complexity of (evaluations of) interventions in the real world.

Compared to other reviews [32, 36–38], similar but also different influencing factors for the implementation of interventions were found on the subcategory level. For example, the subcategory *insufficient time: insufficient time* was also found as barrier for implementation by Naylor et al. [32]. If we consider the aspects reported in this review in the subcategory *insufficient time*, the six articles that reported this barrier indicated four different aspects where *insufficient time* was felt: insufficient time for implementation itself [54, 59], for teachers to participate in trainings regarding the intervention [56], for formal meetings on the intervention [54, 67] and for planning the implementation [54]. The aspects identified by Naylor et al. [32] for *insufficient time* were, for example, lack of time for planning, for training or for notifying parents on family events. These aspects are as various as the aspects identified in this study. For practical application, this means that even though there are consistent results on an aggregated level (*insufficient time*), the underlying aspects can be very diverse.

Influencing factors that have not been identified by similar reviews without the focus on children with a low SES were, for example, that girls felt not enough privacy in the locker rooms [55] or that it requires diverse efforts to achieve parental involvement, for example, language courses, and a personal approach, coffee meeting [57], or a holistic cooperation between the school and parents [66].

No direct comparison has been made between the influencing factors for implementation addressing low SES versus high SES samples, and so it is possible that these influencing factors are not unique to low SES samples. However, it is possible that these influencing factors may be particularly relevant to low SES samples. School buildings and sport facilities, like locker rooms, might be less maintained in underserved areas, leading to a lower feeling of comfort when using them. Furthermore, schools in low SES areas face additional challenges compared to high SES areas, which might negatively affect students’ academic development [79], which might reinforce the cycle between low SES and low child health [15].

The question now arises, which categories or aspects should be considered when developing new or adapting existing interventions for new settings and scaling them up to better reach children with low SES and therefore to contribute in address HI. Every setting and organization has different needs and resources [50]. This is also reflected by the different contexts and characteristics of the interventions identified in this review. The results showed no pattern or influencing factors standing out; however, the results show the breadth of existing influencing factors. Implementation aspects, like influencing factors or implementation outcomes, always depend on the specific intervention, which is being implemented as well as on the setting and context [50, 78]. It is possible that the interventions and their context [72] included in this review were too diverse to find patterns or differences between them. There are recommendations on what to do to decrease HI [1, 76, 80–83], which are partly reflected by the results of this review as well. The intervention materials and personnel conducting the intervention should be culturally appropriate to the target population to build trust, as trust is very important during the whole intervention process [80]. Purposely including strategies on how to reach underserved groups can help addressing HI [1]. The groups that receive interventions, such as those of a low SES, must be involved in the process of implementing effective health-promoting interventions [76]. Participatory approaches can increase the likelihood of successful implementation, and improving the sustainability of interventions and can help balance top-down and bottom-up approaches [82, 83]. These recommendations might seem non-specific, but are worth considering for application in conducting interventions. The intervention mapping approach [84], implementation mapping [35], and the closely related method of co-creation [85] offer guidance for a participatory intervention development and the implementation process.

### School setting—for school staff

All organizations require resources to conduct health-promoting activities. A team for implementation, a health supporting culture, and a head teacher, who supports the intervention are likely to be important factors for successful implementation [86]. These aspects were also reported by articles included in this review in the subcategories *organization* and *communication* in the inner *setting domain*. For example, support from the whole school staff and principal [56, 69], good coordination [54], clear protocols [57], efficient work between stakeholders [69], and clear hierarchical structure [57] and being a small school [59] were reported as facilitators for implementation.

In the following, we would like to give some suggestions for school (head) teachers, school health-promoters, and social workers: Networks and collaboration can facilitate implementation [57, 69]. It is important to be open, persistent and willing to try different things, and to be ready to adjust aspects of the intervention, as each institution has different preconditions, needs, and resources. Working with the community is a promising opportunity, as those collaborations can improve the community networks and benefit school and students [87].

For children outside the school, the family is a very important setting and might be crucial in school-based obesity prevention [88] and is therefore worth considering in implementation activities as well. Regarding school-based nutrition and PA interventions with direct parental involvement (e.g., completing a questionnaire would not count as direct involvement), Verjans-Janssen et al. [89] found mainly positive effects. This may indicate the influence and importance of direct parental involvement in school-based interventions [89], especially in obesity prevention interventions [39] and for children with low SES [90, 91].

Guidelines have been developed for schools on how to implement health-promoting activities [86, 87]. Evidence-informed guidance is of great importance; however, those guidance tend to exhibit a quite theoretical perspective. Building on this foundation, there is still a need for empirical tested and actionable strategies the theory practice translation.

### Methodological considerations—for researcher

Because a wide variety of implementation frameworks exists [92, 93], this review also facilitates standardization and an increase in comparability of results in implementation research in general [94] and for school-based obesity-targeting interventions specifically, using CFIR. CFIR offers several advantages, due to its constant development [95], its method of rating determinants [96], the CFIR outcome addendum [97], and the CFIR-ERIC (Expert Recommendations for Implementation Change: a summary of 73 implementation strategies) matching tool [98, 99].

In identifying and reporting influencing factors using CFIR, very detailed information can be presented, using the categories, as well as more generally by using the domains. This offers comparability on different levels. On the other hand, and this might also be the case for this review, by categorizing all aspects with such many (sub)categories might lead to a reduced applicability for practice. An alternative analytic option for similar investigations would be to conduct an inductive approach for developing a category system using qualitative content analysis and then compare the category system with the CFIR domains and categories. Regarding the field of implementation science, it is rather young [100] and therefore still evolving. This is reflected by, for example, the updated version of CFIR and the various and partly overlapping theories, models, and frameworks [92]. Different terms and definitions are used for similar/the same aspects, for example, fidelity as mentioned above or as evaluated by Schaap et al. [101]. This inconsistency in terms and definitions makes a comparison with existing literature somewhat challenging.

In this review and in many other instances, the application of CFIR is descriptive and linear. This review focused on identifying and evaluating influencing factors for implementation of school-based interventions preventing obesity prevention behaviors for children with low SES. The search term and the eligibility criteria were chosen accordingly. The eligibility criteria of articles was to report on interventions addressing children aged 5 or older, which refers to the youngest age for beginning school [44]. However, this may have led to exclusion of articles on interventions also addressing children entering school before the age of five. There are issues this review could not answer, but future research should address: It can be helpful to quantify the strengths of the influencing factors on the implementation [96, 102], and to analyze which influencing factors are interconnected. It is not only essential to analyze the factors that have an influence on implementation, like this review did, but also on health outcomes [103]. Choosing appropriate implementation strategies [104] and organizing the whole process with an overall evaluation plan [35] should be the standard in implementation evaluation in general and in children with low SES in particular. Furthermore, we want to emphasize the importance of measuring and analyzing different effects of an intervention in groups with different SES [104].

### Limitations

There are several limitations to the results of this review. Every intervention inherently conducts implementation by being performed in real-world settings. The corresponding articles might also report about implementation aspects. Articles in which reported aspects are not labeled as implementation evaluation or process evaluation results though, were not found with the search term and therefore not included in this review, although they might have contained important findings.

All included articles measured the SES on an aggregated school or area level, presumably, because measuring SES for children is challenging and often performed using aggregated proxy measures as described in the “Methods” section [47]. Due to the variety of measuring SES and different available data on an aggregated level, the comparability of included studies might be limited.

Due to a lack of transparency regarding how the influencing factors were identified, influencing factors must have been reported in the empirical results section. Articles reporting influencing factors only as part of the discussion were excluded. In addition, some clustering of influencing factors may not have occurred, because the number of 17 articles in total, six in the group of *simple* interventions, and eleven in the group of *complex* interventions, was too small.

## Conclusion

This review is the first assessing influencing factors for the implementation of interventions promoting obesity prevention behaviors in children with low SES specifically. We identified influencing factors for the implementation of school-based obesity prevention interventions and presented them on a detailed level. This enhances the presentation of results at the most applicable level and contributes to the translation between theory and practice. The detailed reporting shows the tremendous variety of influencing factors for the implementation of obesity prevention interventions for children with low SES. This review could not find striking differences regarding influencing factors for implementation between existing literature without specific target groups and the focus on children with low SES. Still, this review highlights the need of empirical research investigating the processes and dynamics during the adoption, implementation, and sustainment of an intervention as a whole as well as possible differences between groups and settings. Health-promoting interventions for children (with low SES) can lead to less social stratification and can therefore add one piece to the puzzle in the bigger picture of increasing health equity.

### Supplementary Information

**Additional file 1.** PRISMA guidelines for reporting. Checklist of PRISMA guidelines for reporting.

**Additional file 2.** Search strategy. Search strategy and search term used in the different data bases.

**Additional file 3.** List of included articles. Description of included articles, with, e.g., information about article design, data collection and intervention’s description.

**Additional file 4.** Consolidated Framework for Implementation Research Categories with specifications. Consolidated Framework for Implementation Research Categories with specifications made for this review.

**Additional file 5.** Mixed Methods Appraisal Tool ratings. Methodological quality assessment ratings for each article using the Mixed Methods Appraisal Tool items.

**Additional file 6.** Extracted and categorized influencing factors. Extracted and categorized influencing factors for all domains and categories of the Consolidated Framework for Implementation Research Categories.

**Additional file 7.** Most commonly reported (sub)categories by group of simple and complex interventions. The most commonly reported (sub)categories in the group of simple and complex interventions for each CFIR domain.

**Additional file 8.** Number of reported barriers/facilitators and (sub)categories for each CFIR domain. All (sub)categories for each of the five CFIR domains, and all the number of articles reporting the relevant barriers and facilitators.

## Data Availability

The datasets used and/or analyzed during the current study are available from the corresponding author on reasonable request.

## Abbreviations

CFIR: Consolidated Framework of Implementation Research
HI: Health inequity
MMAT: Mixed Methods Appraisal Tool
PA: Physical activity
SES: Socioeconomic status
