# Supplementary material for: Influencing factors for the implementation of school-based interventions promoting obesity prevention behaviors in children with low socioeconomic status: a systematic review
Source: Implement Sci Commun. 2024 Feb 12;5:12. doi: 10.1186/s43058-024-00548-1 (PMC10860312; doi:10.1186/s43058-024-00548-1)
Supplement: Supplementary file 2 — Additional file 2. Search strategy. Search strategy and search term used in the different data bases. [file 43058_2024_548_MOESM2_ESM.docx]

**Search strategy**

Following words will be used in different search term combinations, depending on database

specifications:

|  |  | OR 🡪 | | | | | | |
| --- | --- | --- | --- | --- | --- | --- | --- | --- |
| ***Population*** | *AND 🡪* | kid | child | student | pupil |  |  |  |
| ***Setting*** |  | school | education |  |  |  |  |  |
| ***Intervention*** |  | intervention | program |  |  |  |  |  |
| ***Topic of intervention*** |  | “health promotion” | obesity | “over weight” | overweight | adiposity | “physical activity” | “physical inactivity” |
|  |  | “physical active” | sport | Sitting | Sedentary | “media use” | “screen time” | Nutrition |
|  |  | food | diet |  |  |  |  |  |
| ***Implementation outcome*** |  | adoption | dissemination | adaption | implementation | sustainability | maintenance | “process evaluation” |
| ***Socio-economic status*** |  | socioeconomic | socio-economic | income |  |  |  |  |

**SCOPUS**

TITLE-ABS-KEY(kid OR child OR student OR pupil)AND TITLE-ABS-KEY(*school* OR educat*)AND TITLE-ABS-KEY(interven* OR program*)AND TITLE-ABS-KEY("health promotion" OR obes* OR "over weight" OR overweigh* OR adipos* OR "physical *activ*" OR sport OR sit* OR sedentar* OR "media use" OR "screen time" OR nutrition OR food OR diet*)AND TITLE-ABS-KEY (adopt* OR disseminat* OR adapt* OR implement* OR sustain* OR maintain* OR "process evaluation") AND (*income
OR socioeconom*)

Filter: “Articles”

Date: 24.06.21

Update Search: 29.03.2023

**PUBMED**

(((((kid[Title/Abstract] OR kids[Title/Abstract] OR child*[Title/Abstract] OR student*[Title/Abstract] OR pupil*[Title/Abstract]) AND (*school*[Title/Abstract] OR educat*[Title/Abstract])) AND (interven*[Title/Abstract] OR program*[Title/Abstract])) AND ("health promotion"[Title/Abstract] OR obes*[Title/Abstract] OR "over weight"[Title/Abstract] OR overweigh*[Title/Abstract] OR adipos*[Title/Abstract] OR "physical inactiv*"[Title/Abstract] OR "physical activ*"[Title/Abstract] OR sport*[Title/Abstract] OR sit[Title/Abstract] OR sitting[Title/Abstract] OR sedentar*[Title/Abstract] OR "media use"[Title/Abstract] OR "screen time"[Title/Abstract] OR nutrition[Title/Abstract] OR food[Title/Abstract] OR diet*[Title/Abstract])) AND (adopt*[Title/Abstract] OR disseminat*[Title/Abstract] OR adapt*[Title/Abstract] OR implement*[Title/Abstract] OR sustain*[Title/Abstract] OR maintain*[Title/Abstract] OR "process evaluation"[Title/Abstract])) AND (socioeconom* OR socio-econom* OR income OR "low income" OR "low-income")

Filter: None

Date: 24.06.21

Update Search: 29.03.2023

**ERIC, SportDis, PsychArticles, Education Source, SocINDEX via EBSCO:**

(((AB ((kid* OR child* OR student* OR pupil*))) OR (TI ((kid* OR child* OR student* OR pupil*)))) AND ((TI ((school* OR educat*))) OR (AB ((school* OR educat*)))) AND ((TI ((interven* OR program*))) OR (AB ((interven* OR program*)))) AND ((TI (("health promotion" OR obes* OR "over weight" OR overweigh* OR apidos* OR "physical *activ*" OR sport* OR sit* OR sendetar* OR "media use" OR "screen time" OR nutrition OR food OR diet*))) OR (AB (("health promotion" OR obes* OR "over weight" OR overweigh* OR apidos* OR "physical *activ*" OR sport* OR sit* OR sendetar* OR "media use" OR "screen time" OR nutrition OR food OR diet*)))) AND ((TI ((adopt* OR disseminat* OR adapt* OR implement* OR sustain* OR maintain* OR "process evaluation"))) OR (AB ((adopt* OR disseminat* OR adapt* OR implement* OR sustain* OR maintain* OR "process evaluation")))) AND (TX ((socioeconom* OR socio-econom* OR income OR "low income" OR "low-income"))))

Filter: “Article” or “Jorunal Article”

Date: 01.07.21

Update Search: 29.03.2023
