# Supplementary figures and images for: Influencing factors for the implementation of school-based interventions promoting obesity prevention behaviors in children with low socioeconomic status: a systematic review
Source: Implement Sci Commun. 2024 Feb 12;5:12. doi: 10.1186/s43058-024-00548-1 (PMC10860312; doi:10.1186/s43058-024-00548-1)

## Slide 1
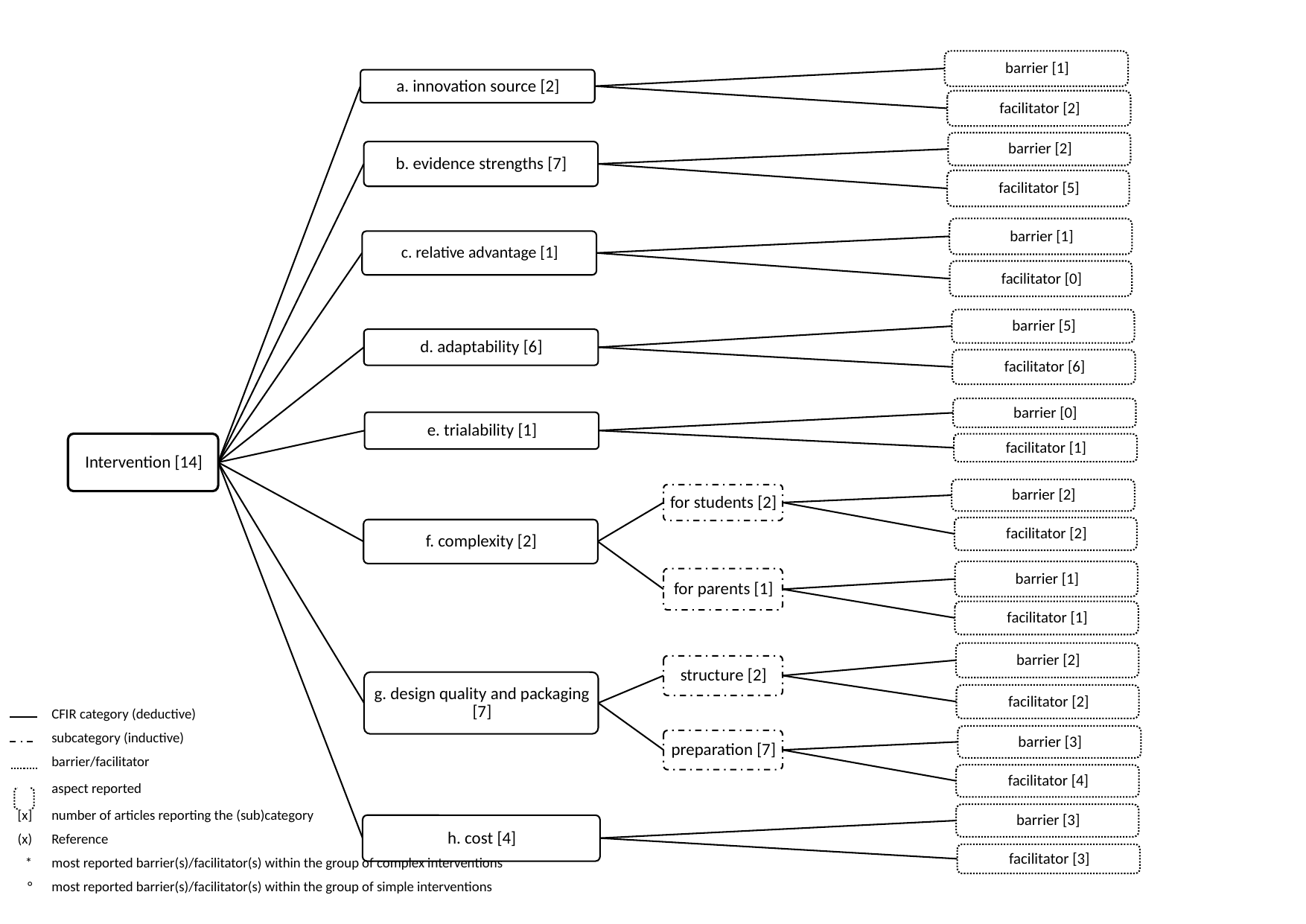

## Slide 2
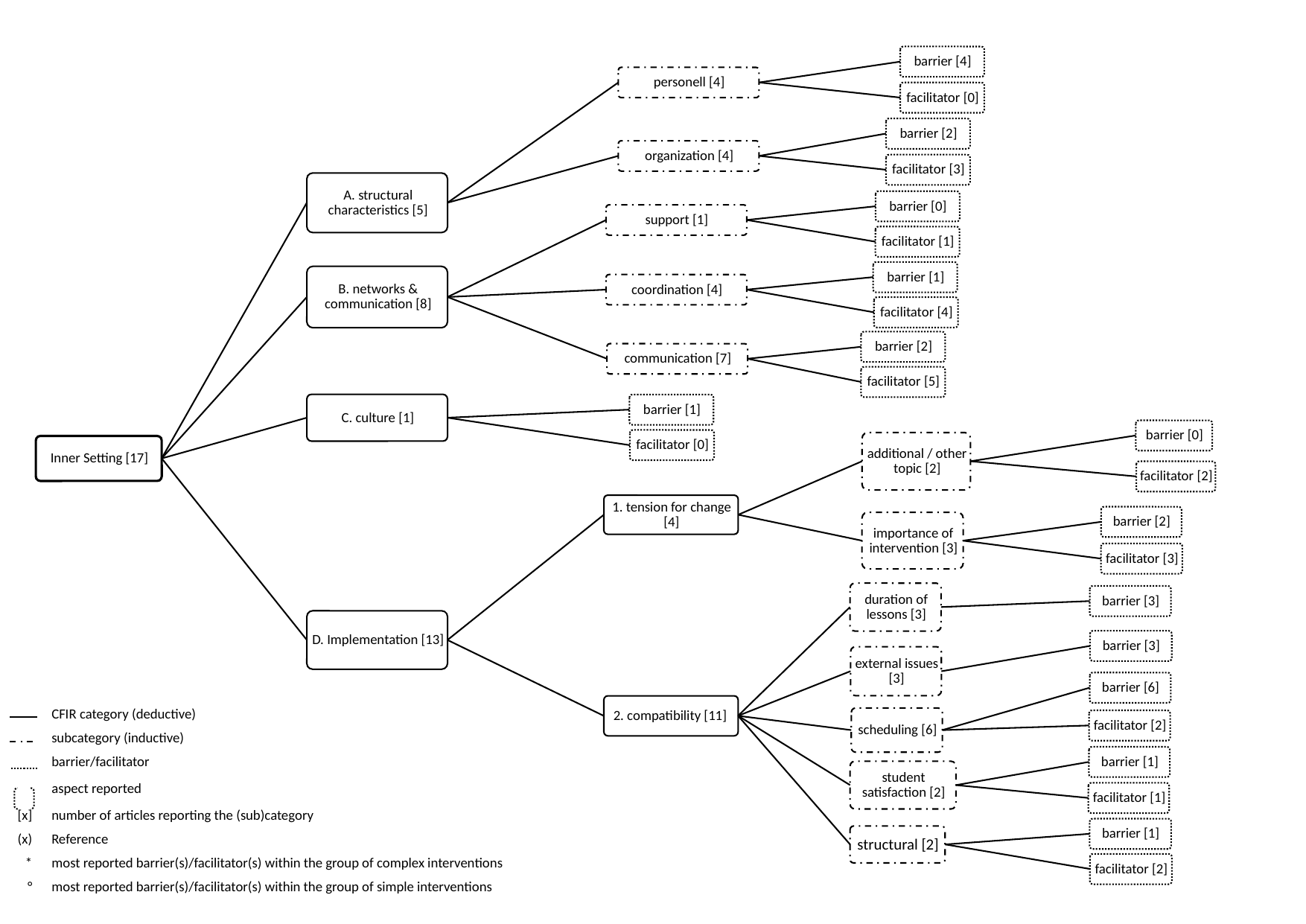

## Slide 3
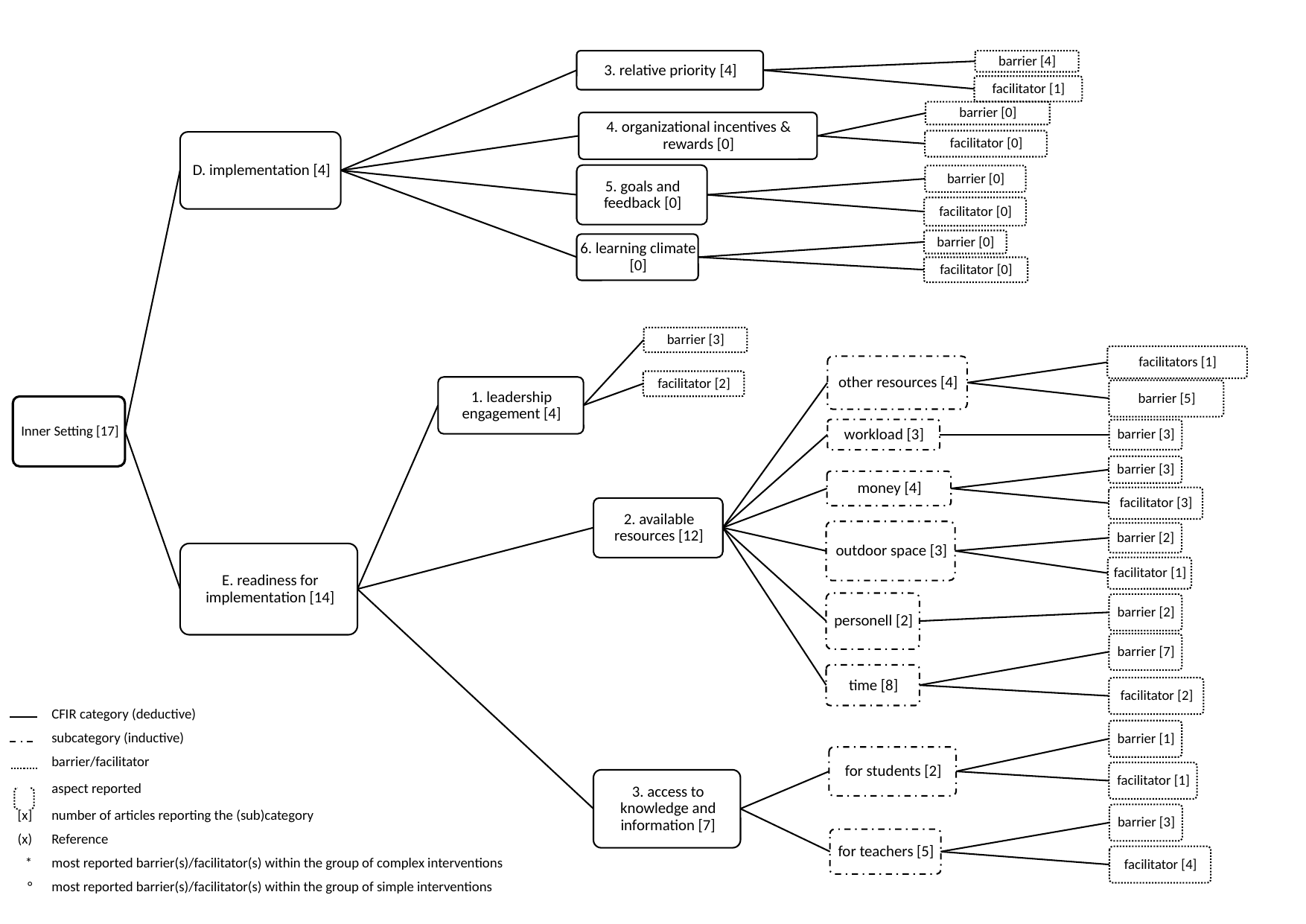

## Slide 4
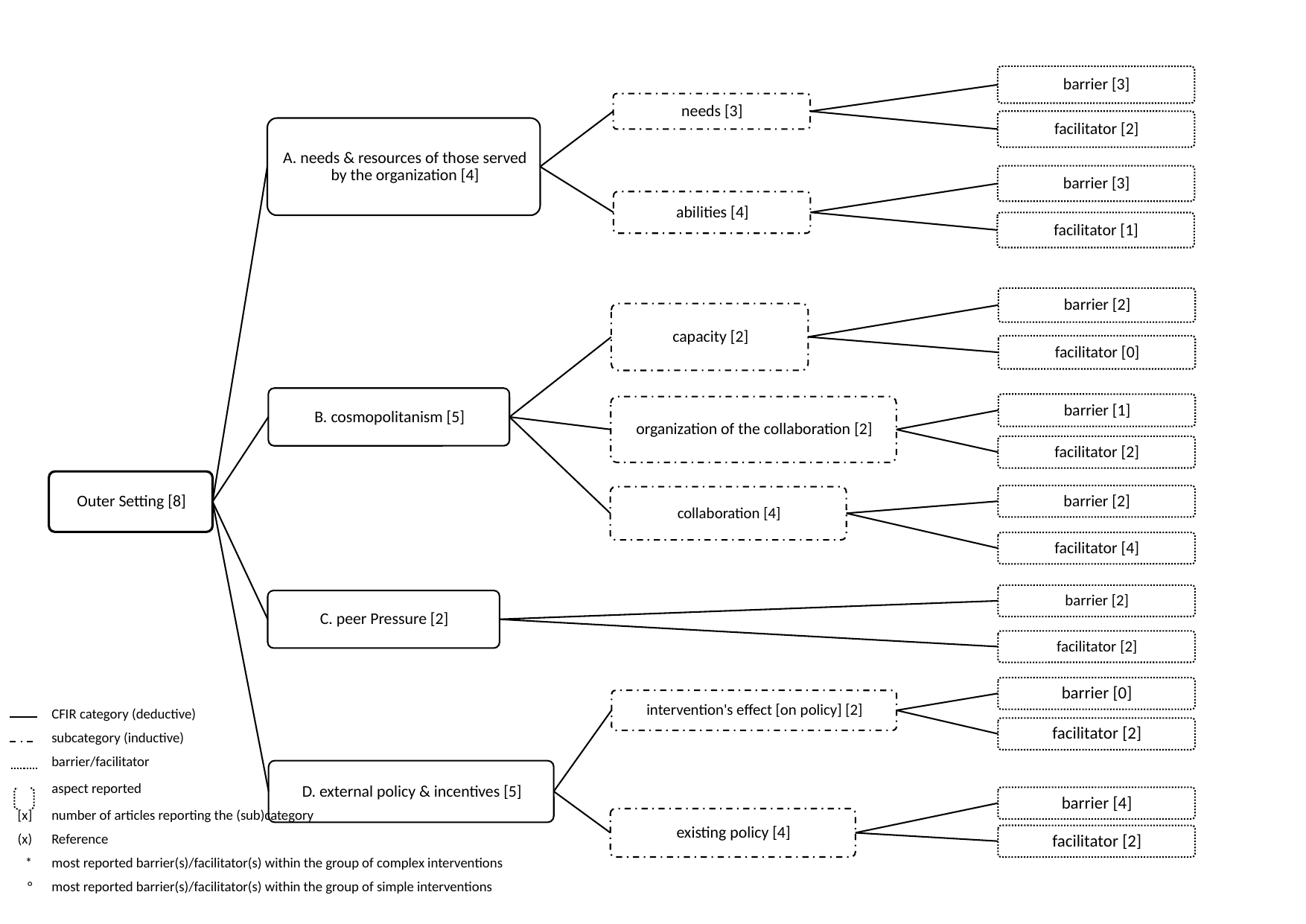

## Slide 5
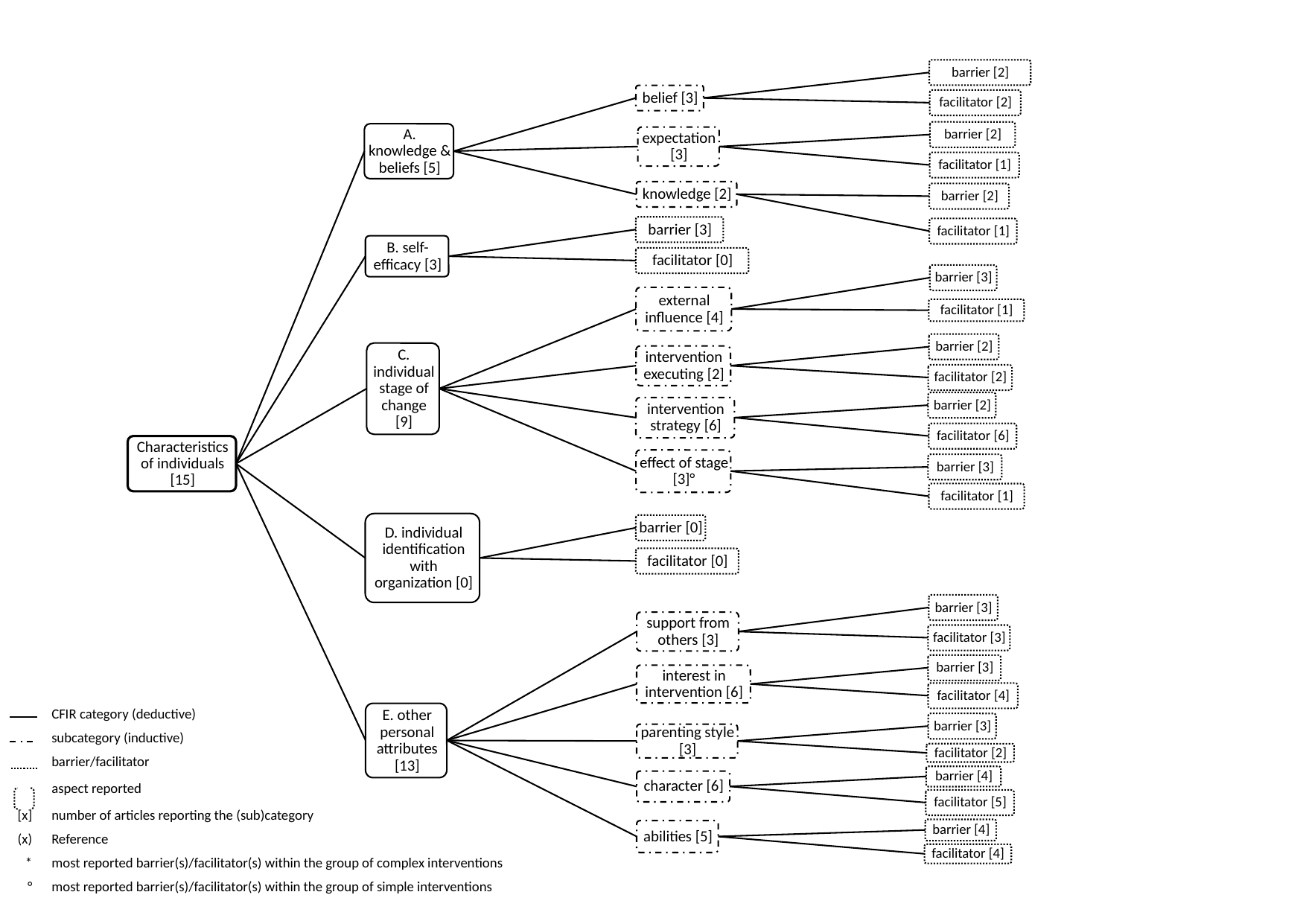

## Slide 6
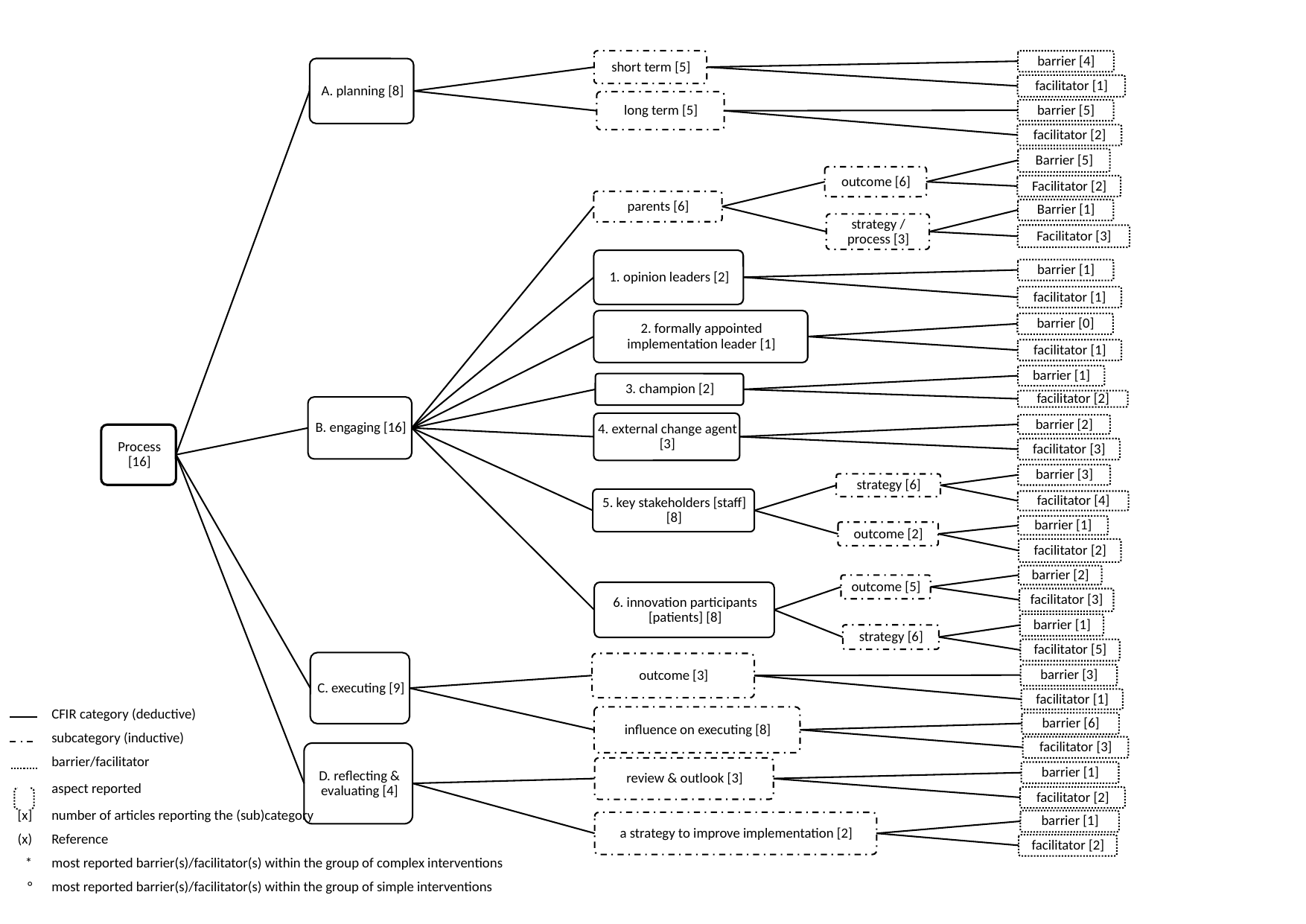

Supplement: Supplementary file 8 — Additional file 8. Number of reported barriers/facilitators and (sub)categories for each CFIR domain. All (sub)categories for each of the five CFIR domains, and all the number of articles reporting the relevant barriers and facilitators. [file 43058_2024_548_MOESM8_ESM.zip › Additional file 8/Additional_file_8_(sub)cats_bar_fac_05.12R2.pptx]

## Slide 1
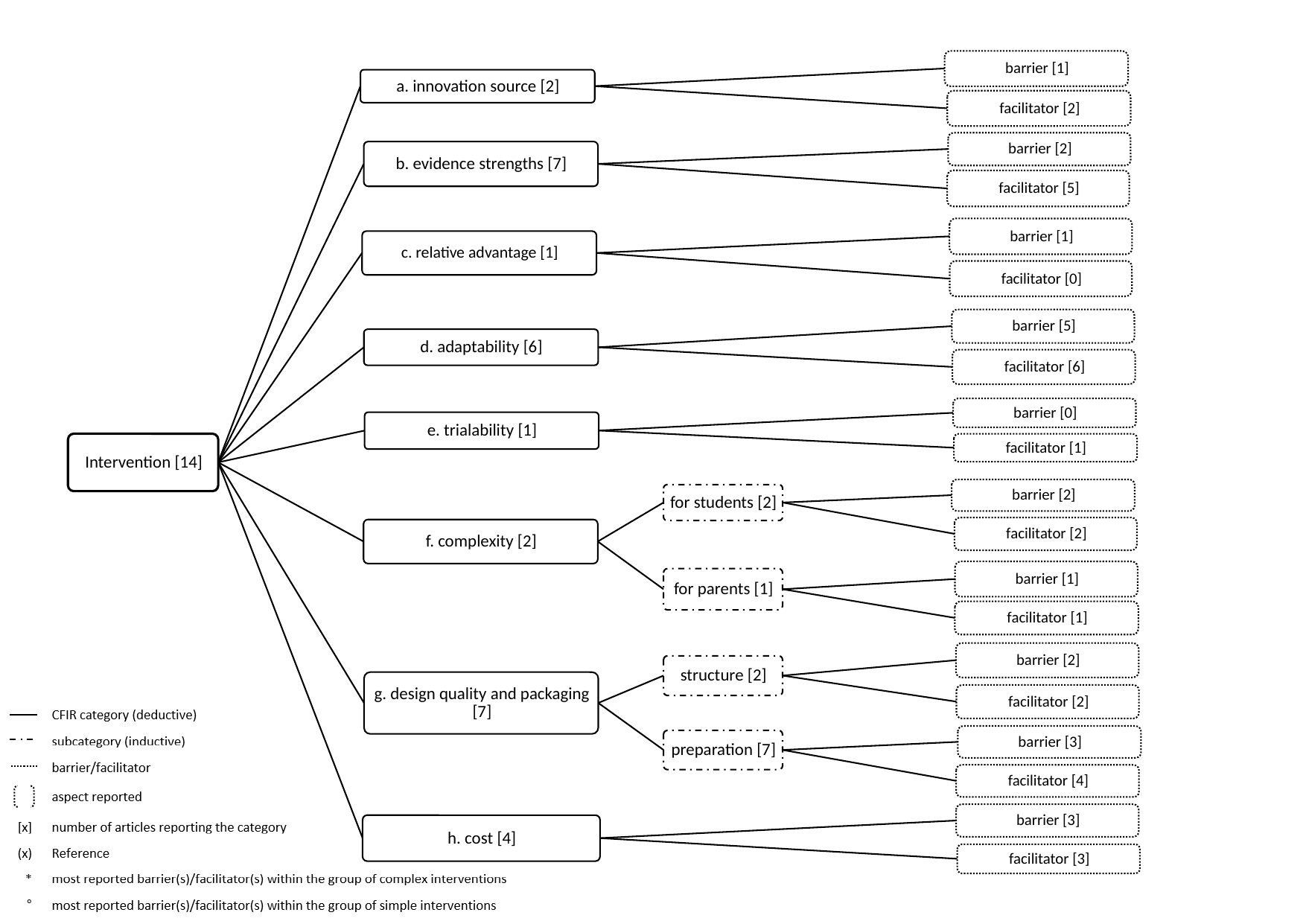

## Slide 2
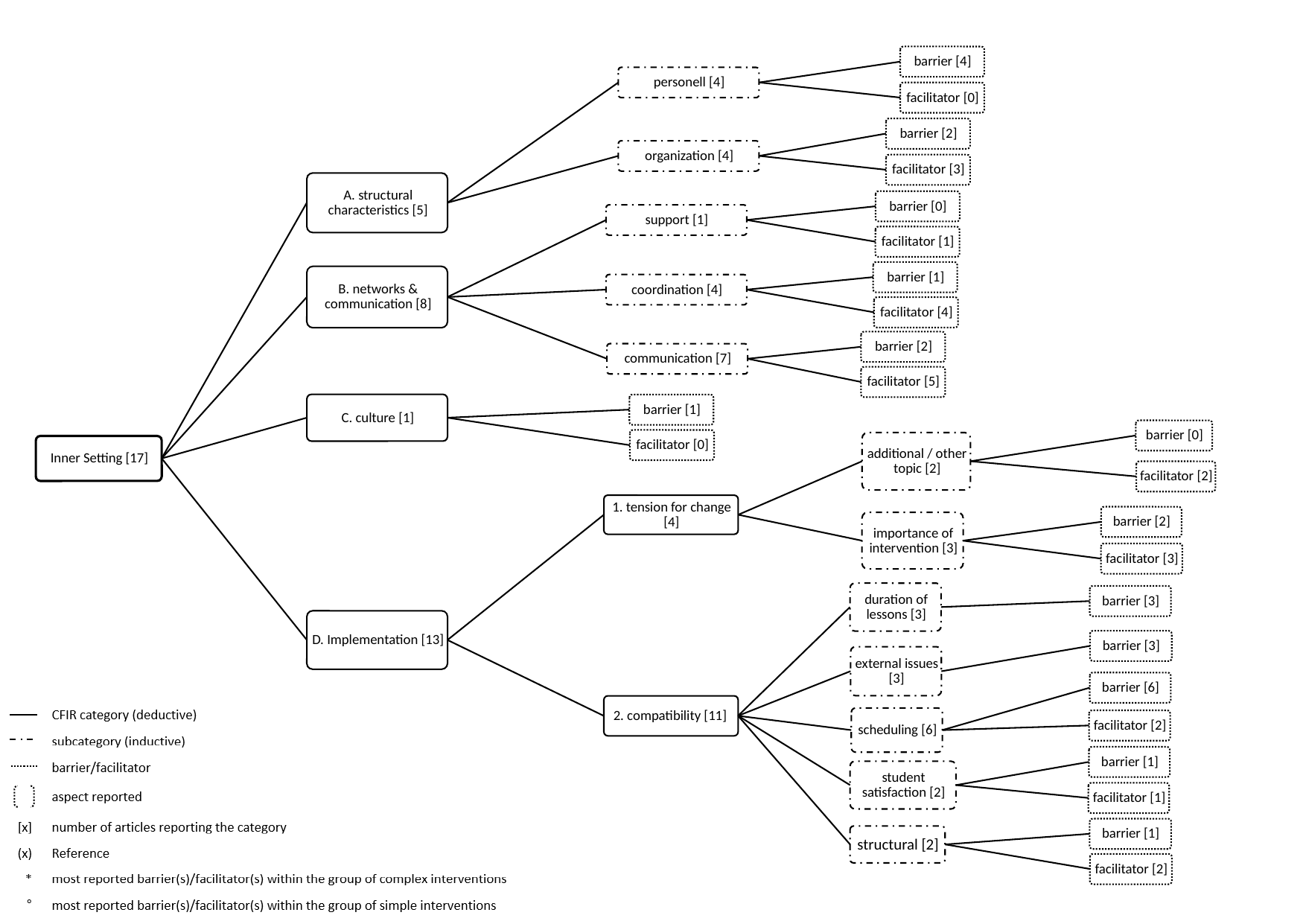

## Slide 3
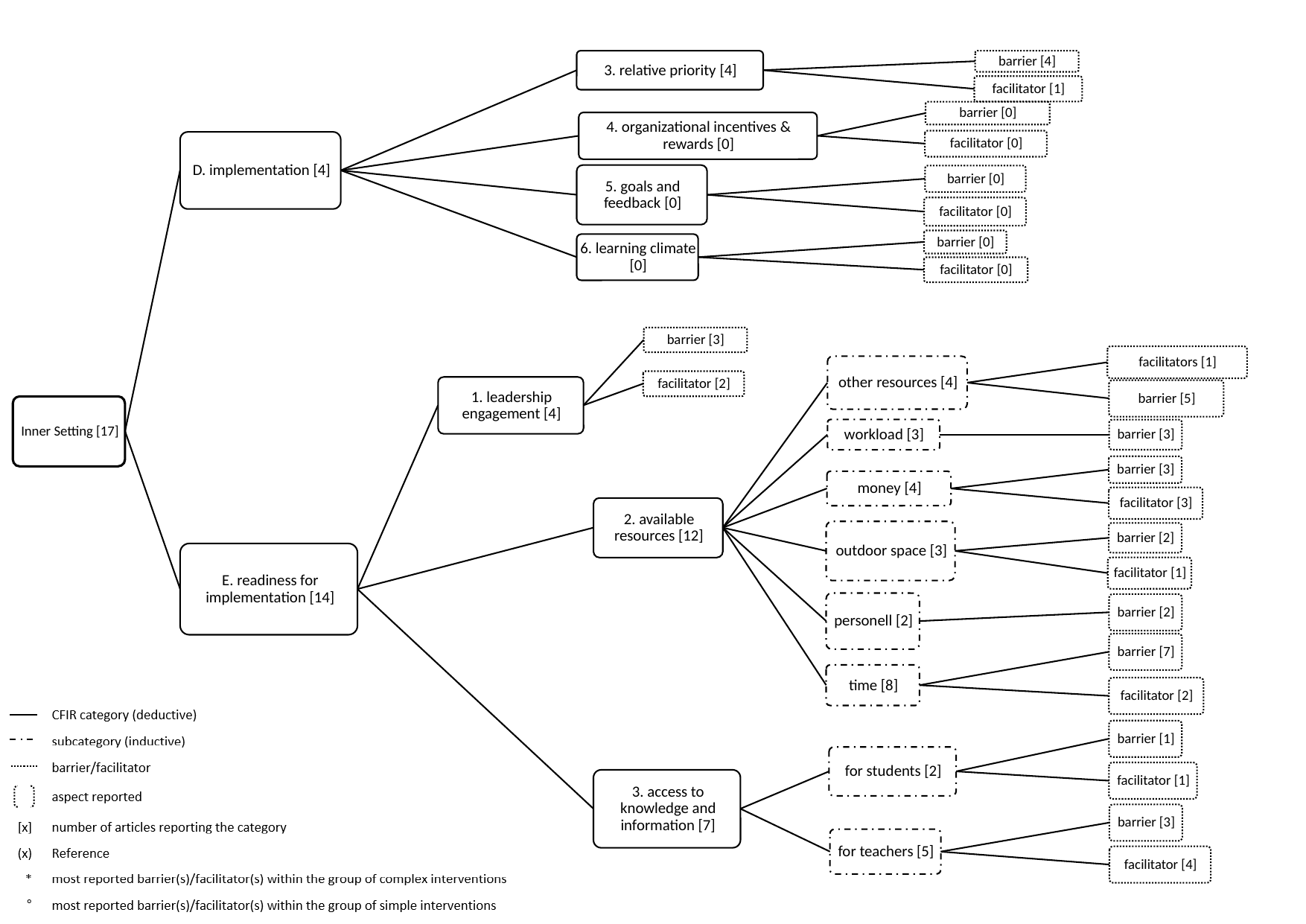

## Slide 4
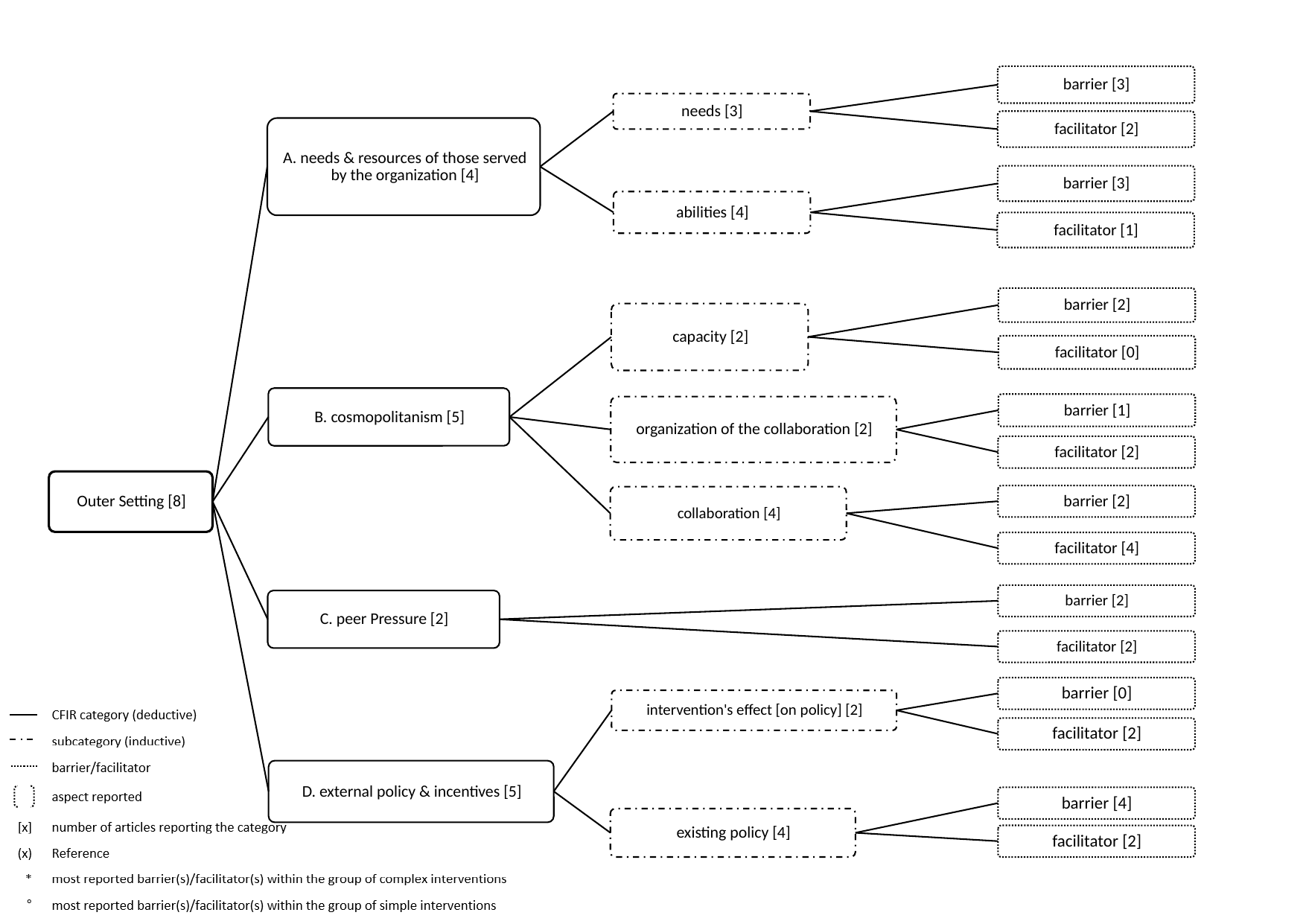

## Slide 5
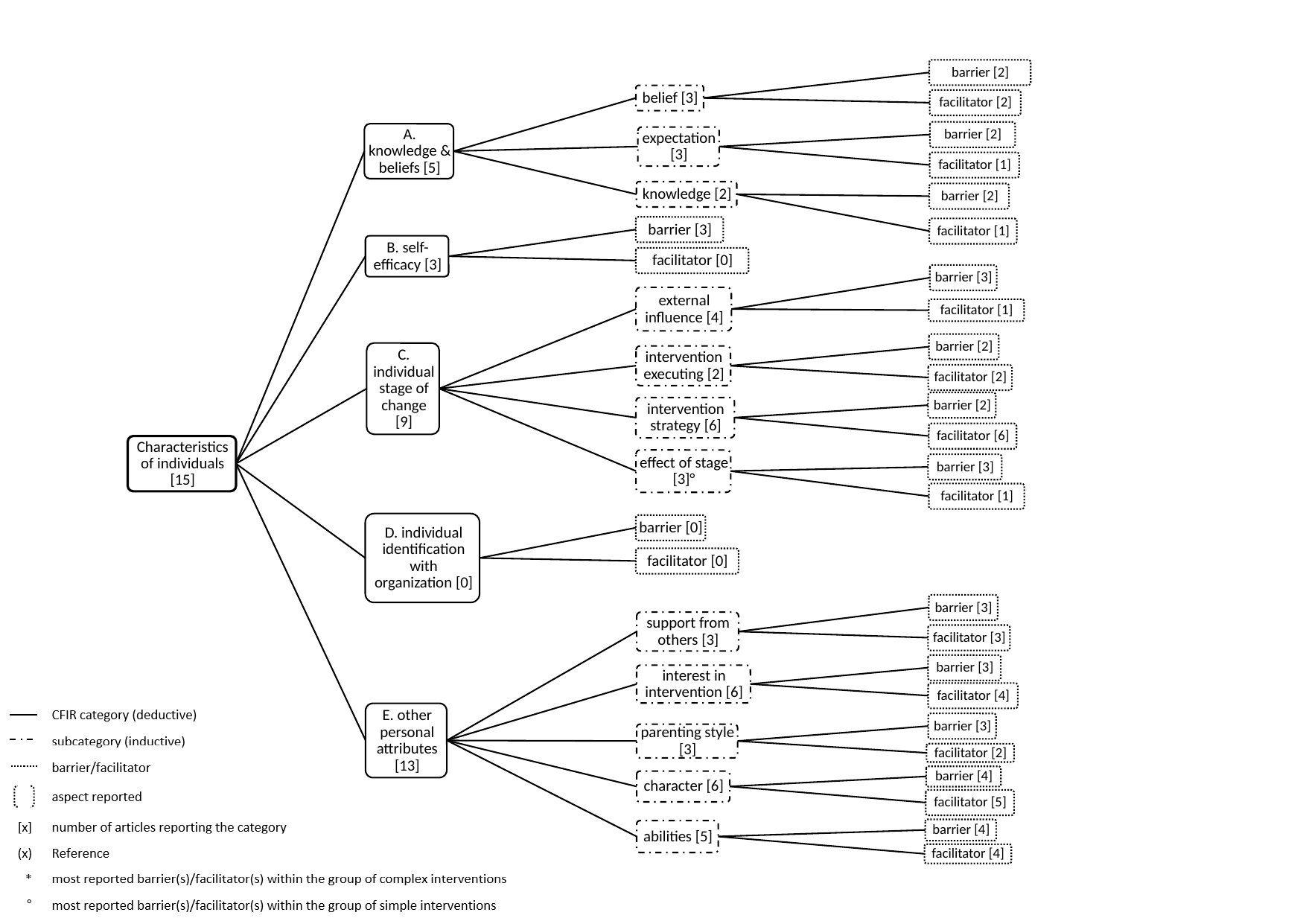

## Slide 6
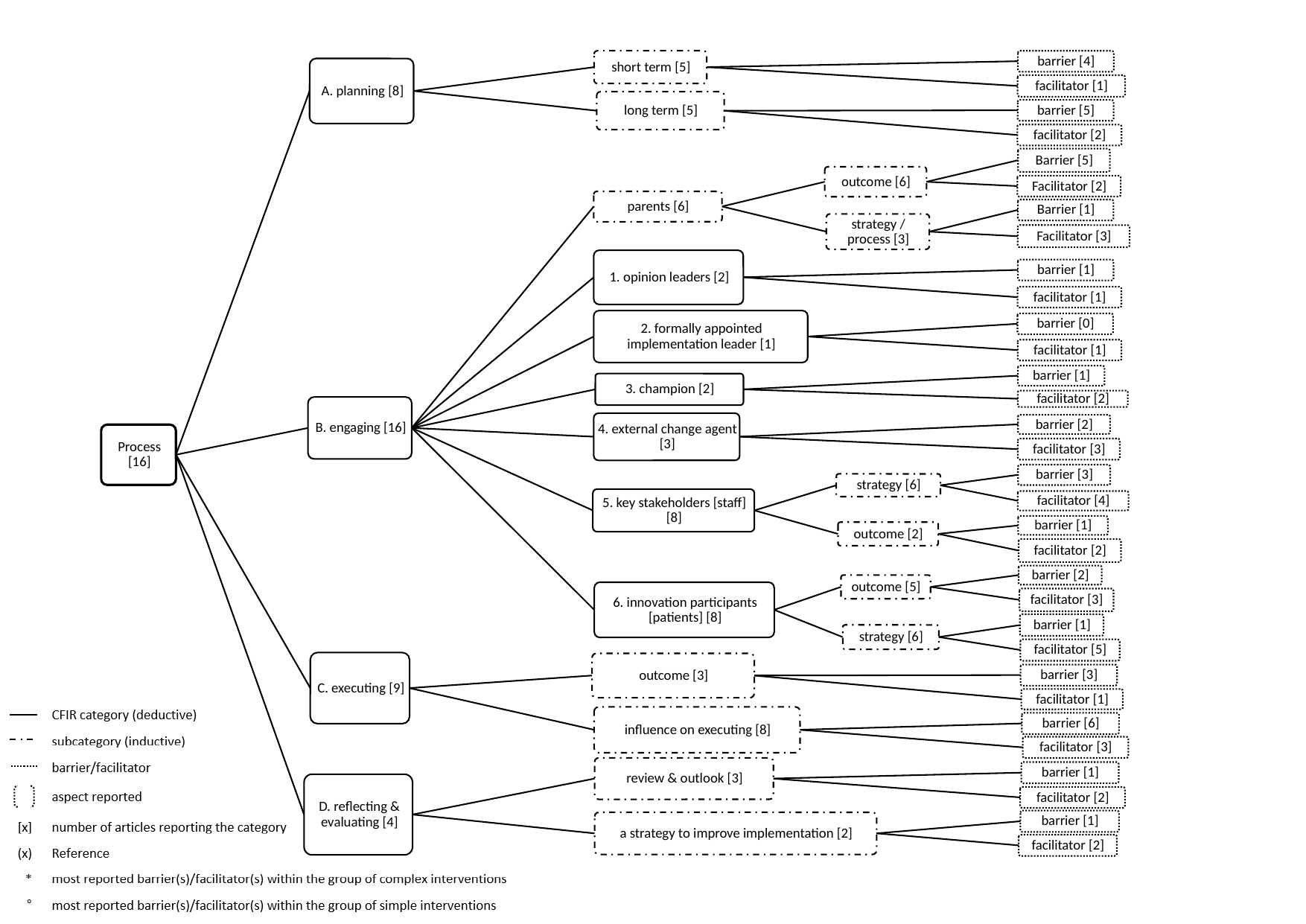

Supplement: Supplementary file 8 — Additional file 8. Number of reported barriers/facilitators and (sub)categories for each CFIR domain. All (sub)categories for each of the five CFIR domains, and all the number of articles reporting the relevant barriers and facilitators. [file 43058_2024_548_MOESM8_ESM.zip › Additional file 8/Additional_file_8_(sub)cats_bar_facR2.pptx]
